# Nitrogen-Containing Fabric Adsorbents Prepared by Radiation Grafting for Removal of Chromium from Wastewater

**DOI:** 10.3390/polym10070744

**Published:** 2018-07-05

**Authors:** Natsuki Hayashi, Jinhua Chen, Noriaki Seko

**Affiliations:** 1 Department of Advanced Functional Materials Research, Takasaki Advanced Radiation Research Institute, National Institutes for Quantum and Radiological Science and Technology (QST), 1233 Watanuki-machi, Takasaki, Gunma 370-1292, Japan; hayashi.natsuki@qst.go.jp (N.H.); seko.noriaki@qst.go.jp (N.S.); 2 School of Science and Technology, Gunma University, 1-5-1 Tenjin, Kiryu, Gunma 376-8516, Japan

**Keywords:** radiation grafting, adsorbents, wastewater, chromium removal, adsorption capacity, amidoximation

## Abstract

To remove chromium from wastewater effectively, two types of nitrogen-containing fabric adsorbents, having amidoxime ligand groups and quaternary ammonium anion exchange groups, respectively, were prepared by radiation grafting. In brief, the amidoxime adsorbent is obtained by grafting of acrylonitrile (AN)/methacrylic acid (MAA) onto a nonwoven fabric and subsequent amidoximation with hydroxylamine, while the ammonium adsorbent is obtained by grafting of chloromethylstyrene (CMS) followed by quaternization with trimethylamine. The AN/MAA-grafting reaches a high degree of grafting more than 100%, and the resulting amidoxime adsorbent reaches a high amidoxime density of 4.53 mmol/g. On the other hand, the CMS-grafting reaches a much higher degree of grafting above 200%, and the resulting ammonium adsorbent reaches a high ammonium density of 3.51 mmol/g. FTIR/ATR and TGA/DTA are used for the characterization of the grafted fabrics as well as the relevant fabric adsorbents. Furthermore, the chromium removal of the prepared fabric adsorbent is tested in both batch and column modes. It has been confirmed that the chromium removal was largely dependent on the pH of the solution. At pH 5.0, the amidoxime adsorbent shows a high Cr(III) adsorption capacity of 31.68 mg/g, while the ammonium adsorbent shows a much higher Cr(VI) adsorption capacity of 130.65 mg/g.

## 1. Introduction

Chromium compounds are widely used in industrial processes, and the wastewater from these industries contains both trivalent Cr(III) and hexavalent Cr(VI) ions. The Cr(VI) is a strong oxidant, and is recognized to be much more toxic than the Cr(III) [1]. The maximum permissible limit for chromium content in drinking water recommended by the World Health Organization (WHO) is 0.05 ppm. Chemical precipitation [2], electrochemical reduction and coagulation [3], membrane separation [4], ion exchange [5,6] and adsorption [7,8] are used for removing chromium from wastewater. Among these methods, adsorption is effective for the chromium removal [9]. However, the traditional adsorption technique that uses particles, such as active carbon, and biomaterials as the adsorbents requires additional separation process [9,10,11,12,13]. Recently, adsorbents in the form of fiber and fabric are used wherein the adsorbent/wastewater separation process can be eliminated. Furthermore, these adsorbents are easily regenerated and the desorbed metal ions can be recycled [14,15,16,17].

In this work, we modified an existing nonwoven fabric for chromium removal from wastewater. The nonwoven fabric produced from polyethylene-coated polypropylene (PE/PP) fibers has a large surface area compared to active carbon and particles. The PE/PP fabric was modified by radiation grafting and subsequent chemical treatment, where the nitrogen-containing functional groups, amidoxime and quaternary ammonium groups were introduced into the fabrics [18,19,20,21,22]. Herein, the radiation grafting is a very attractive method that can introduce functional groups into the interior of solid materials as well as on the surface [23,24]. By using radiation grafting, hydrophobic polymeric films, such as polytetrafluoroethylene (PTFE) [25], poly(ethylene-*co*-tetrafluoroethylene) (ETFE) [26], poly(vinylidene fluoride) (PVDF) [27] and polyetheretherketone (PEEK) [28], could be converted to hydrophilic polymer electrolyte membranes for fuel cells, and polyolefin fibers and fabrics could be modified as adsorbents for removal of heavy metal ions from water [29,30,31,32,33,34].

The PE/PP nonwoven fabric was first electron-beam irradiated by an electron accelerator. The stable polymeric radicals were generated in the fabrics during the irradiation [19,35]. The irradiated fabric was then immersed in an oxygen-free monomer solution at the designed temperature for grafting, under which the polymeric radicals initiated the graft polymerization. In this work, an amidoxime fabric adsorbent was synthesized by co-grafting of acrylonitrile (AN) and methacrylic acid (MAA) and subsequent amidoximation with hydroxylamine [36,37], while an ammonium fabric adsorbent was synthesized by grafting of chloromethylstyrene (CMS) and subsequent quaternization with trimethylamine [38]. The chromium removal of the resultant fabric adsorbents was tested in batch and column modes.

## 2. Experimental

### 2.1. Materials

**Scheme 1:**
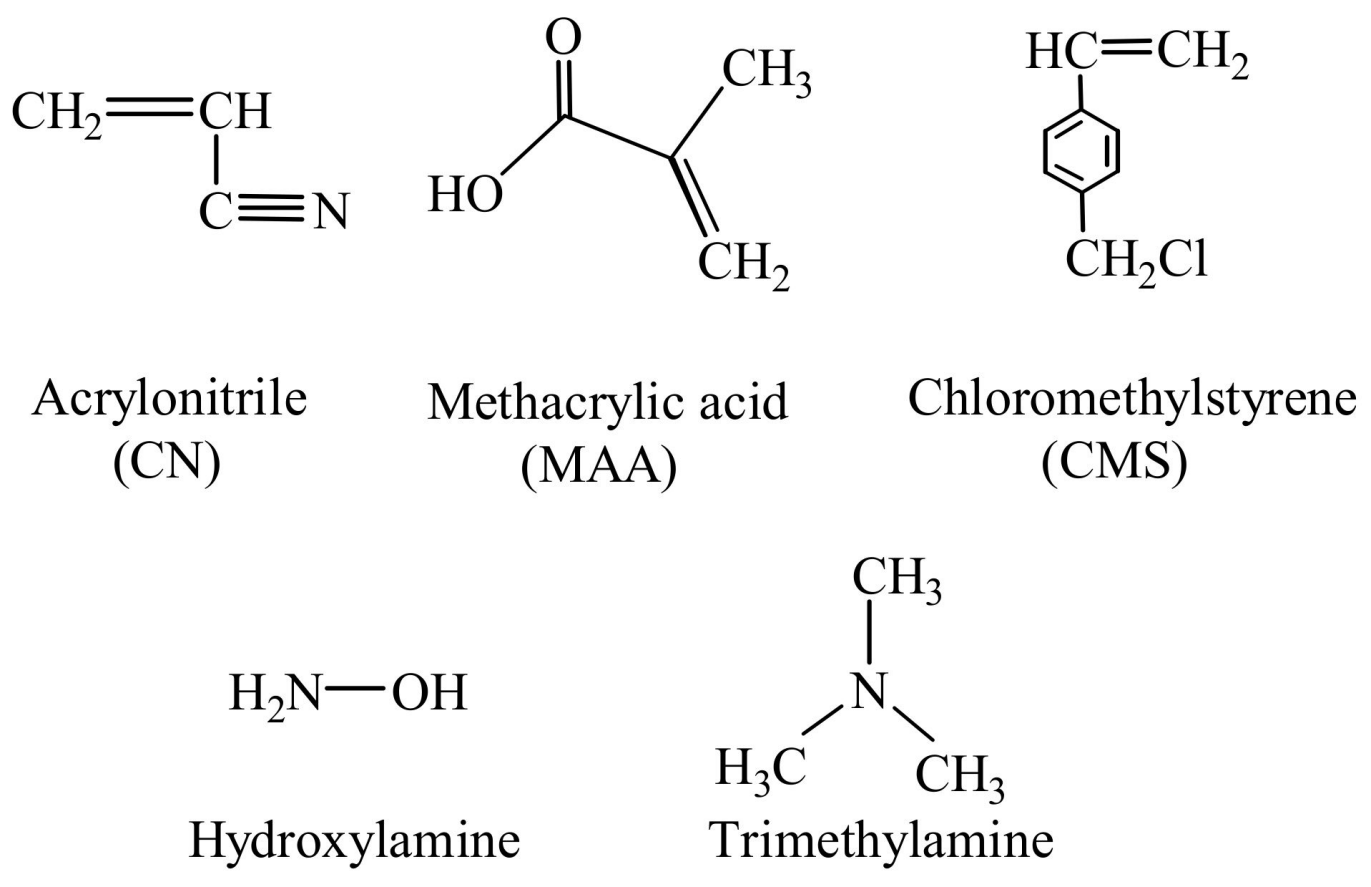
Chemical structures of the monomers and chemical reagents used in this study.

The PE/PP nonwoven fabric with a thickness of about 100 μm was supplied by Kurashiki Textile Manufacturing Co., Ltd. (Osaka, Japan). The diameter of PP/PE fiber was about 13 µm. The important chemical agents used in this study are shown in Scheme 1. The monomers of AN and MAA were obtained from Kanto Chemical Co., Inc. (Tokyo, Japan), and were used without any further treatment. The CMS was purchased from AGC Seimi Chemical Co., Ltd. (Kanagawa, Japan). The CMS monomer was purified by activated alumina to remove the *tert*-butylcatechol inhibitor just before use [38]. The hydroxylamine hydrochloride (NH_2_OH·HCl) and trimethylamine, as well as the Cr(III) and Cr(VI) standard solutions (1000 ppm), were also obtained from Kanto Chemical Co., Inc. (Tokyo, Japan). Ultra-pure water was used throughout the work. All the other materials and chemicals were of analytical grade and were used as received. The mixture solvent of methanol and water in the weight ratio of 1:1 was used for the preparation of the hydroxylamine and trimethylamine solutions. The potassium hydroxide (KOH), sodium hydroxide (NaOH), and nitric acid (HNO_3_) were used for the pH adjustment.

### 2.2. Radiation Grafting

The fabric in the size of 3.0 cm × 3.0 cm was placed in a vacuum packaged polyethylene bag for electron beam irradiation at a low acceleration voltage of 250 kV and an electric current of 2.6 mA. The irradiated fabric was transferred into a glass ampoule containing an oxygen-free monomer solution for grafting at 40 °C. The AN/MAA/DMSO solution in the weight ratio of 35/15/50 was used for the co-grafting of AN/MAA [36], and the emulsion containing 3.0 wt % CMS, 0.3 wt % Tween 20 (an emulsifying agent) and 96.7 wt % water was used for the grafting of CMS [38]. After grafting, the fabric was taken out from the ampoule and was washed with dimethylformamide (DMF) for several times, and was then dried in vacuum at 60 °C for more than 24 h. The degree of grafting was calculated as:

Degree of grafting (%) = (*W_g_* − *W_o_*)/*W_o_* × 100 (1)

where *W_o_* and *W_g_* are the dry weights of the fabric before and after the grafting, respectively.

### 2.3. Functionalization

The amidoxime fabric adsorbent was obtained by treatment of the AN/MAA-grafted fabric with a hydroxylamine solution [36,37], that is, the AN/MAA-grafted fabric was immersed in a 3.0 wt % hydroxylamine solution (pH 7.0 adjusted by KOH) at 80 °C for more than 15 min. Under this condition, it was confirmed that all of the cyanide (CN) groups on the graft chains were converted into amidoxime (C(NH_2_)=NOH) groups [36]. On the other hand, the ammonium fabric adsorbent was prepared by immersing the CMS-grafted fabric in a 0.25 M trimethylamine solution at 80 °C for 30 min, where the chloromethyl (CH_2_Cl) groups on the graft chains were converted into quaternary ammonium (N(CH_3_)_3_Cl) groups [38,39]. The density of functional groups of the adsorbents was calculated as:

Density of functional groups (mmol/g) = (*W_a_* − *W_g_*)/(*MW_a_*) × 1000 (2)

where *W_a_* and *W_g_* are the dry weights of the adsorbent and the relevant grafted fabric, respectively, and *M* is the increased molecular weight of the sample after the functionalization. For the amidoxime adsorbent, it was 33, and for the ammonium adsorbent, it was 59.

### 2.4. Fourier Transform Infrared (FTIR) and Thermogravimetry/Differential Thermal Analysis (TG/DTA) Measurements

The nonwoven fabrics, grafted fabrics and the resultant fabric adsorbents were characterized by FTIR spectroscopy in attenuated total reflectance (ATR) mode (PerkinElmer Japan Co., Ltd., Yokohama, Japan). Prior to the FTIR measurements, the samples were sandwiched with ETFE films and hot-pressed at 130 °C for 2 min to obtain good surface for the ATR probe. The samples were analyzed with a resolution of 4 cm^−1^ and 64 scans in the range of 450–4000 cm^−1^. Thermo Plus2/TG-DTA (Rigaku Corp., Tokyo, Japan) was used for the TG/DTA analysis of the fabric adsorbents. The specimen (about 8 mg) was heated from room temperature to 550 °C at a heating rate of 10 °C/min with the nitrogen gas flow rate of 100 mL/min.

### 2.5. Batch and Column Mode Adsorptions

**Figure 1:**
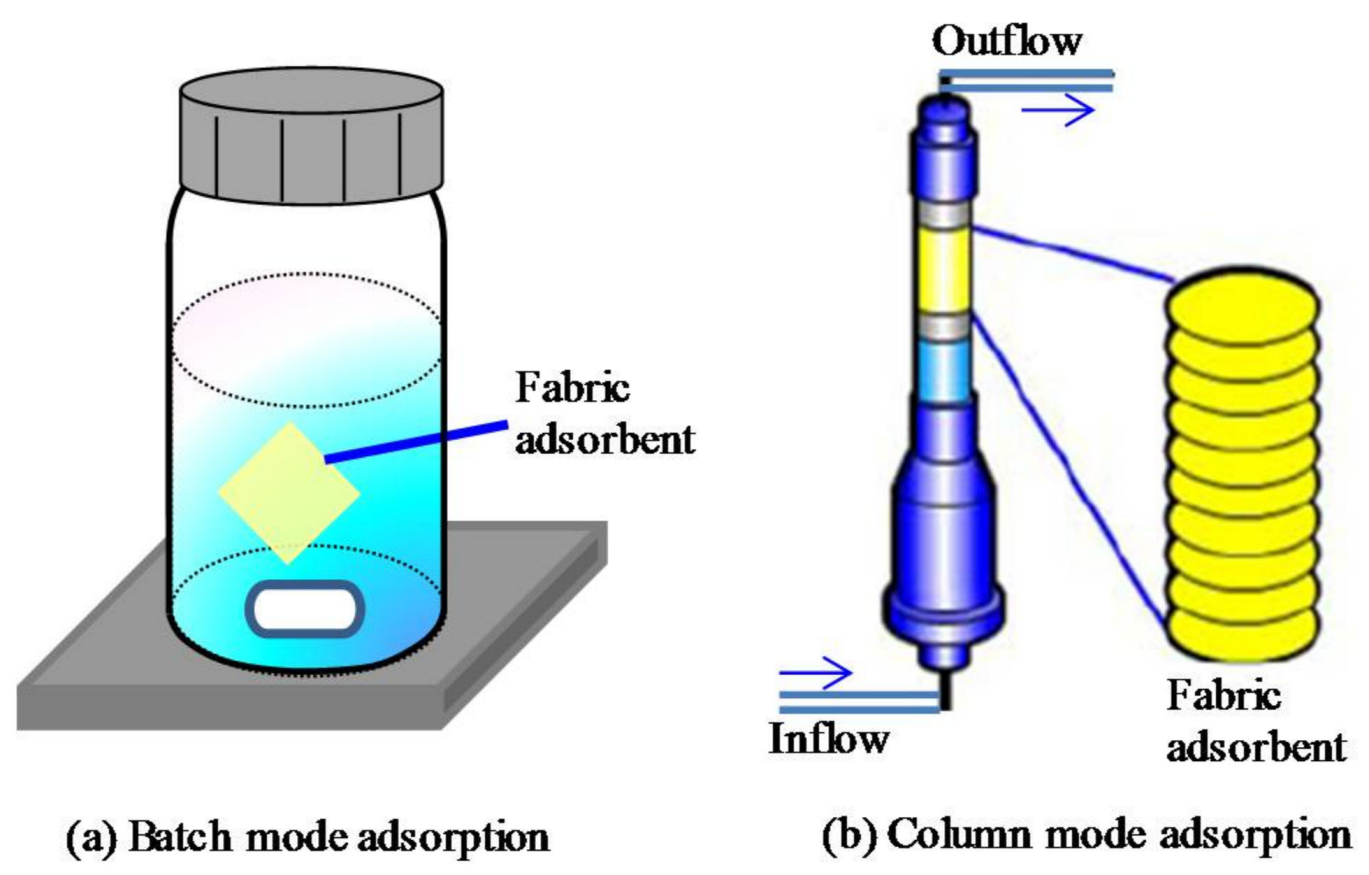
Batch and column mode adsorptions.

The batch and column mode adsorptions for the chromium removal test were shown in Figure 1. The Cr(III) and Cr(VI) solutions were prepared by diluting the corresponding standard solutions with ultra-pure water. The pH of the solutions was adjusted using nitric acid (HNO_3_) and sodium hydroxide (NaOH). The chromium concentrations in the solution before and after the adsorption were detected by an Inductively Coupled Plasma-Optical Emission Spectrometer (ICP-AES, Optima 8300, PerkinElmer Japan Co., Ltd., Yokohama, Japan).

For the bath mode adsorption, about 20 mg of the fabric adsorbent was immersed in 45 mL of the chromium solution and was stirred for 90 min at room temperature. Here, in terms of stoichiometry, the amount of the functional groups in the fabric adsorbent was much greater than the chromium ions in the solution. The adsorptions were also performed for 24 h, and similar results were obtained, indicating that the adsorption for 90 min was sufficient to achieve the equilibrium. The chromium removing (%) was calculated as:

Chromium removing (%) = (*C_o_* − *C_i_*)/*C_o_* × 100 (3)

where *C_o_* and *C_i_* are the chromium concentration before and after the immersion, respectively.

For the column mode adsorption, the fabric adsorbent (about 60 mg) was filled into a column and equilibrated in water for 24 h before the test. The inner diameter of the column was 7 mm, and the filled volume was about 0.1 mL. The chromium solution was flowed through the column at a rate of 60 mL/h. The chromium concentration of the solution collected from the outflow was analyzed by the ICP-AES, and the chromium removing (%) was calculated using Equation (3). Furthermore, the chromium adsorption capacity of the relevant adsorbent was calculated as:

Adsorption capacity (mg/g) = *A_d_*/*W_a_* (4)

where *A_d_* is the chromium adsorbed amount of the adsorbent, which is obtained from the relationship between chromium removing (%) and flow time.

## 3. Results and Discussion

### 3.1. Preparation of the Fabric Adsorbents

**Scheme 2:**
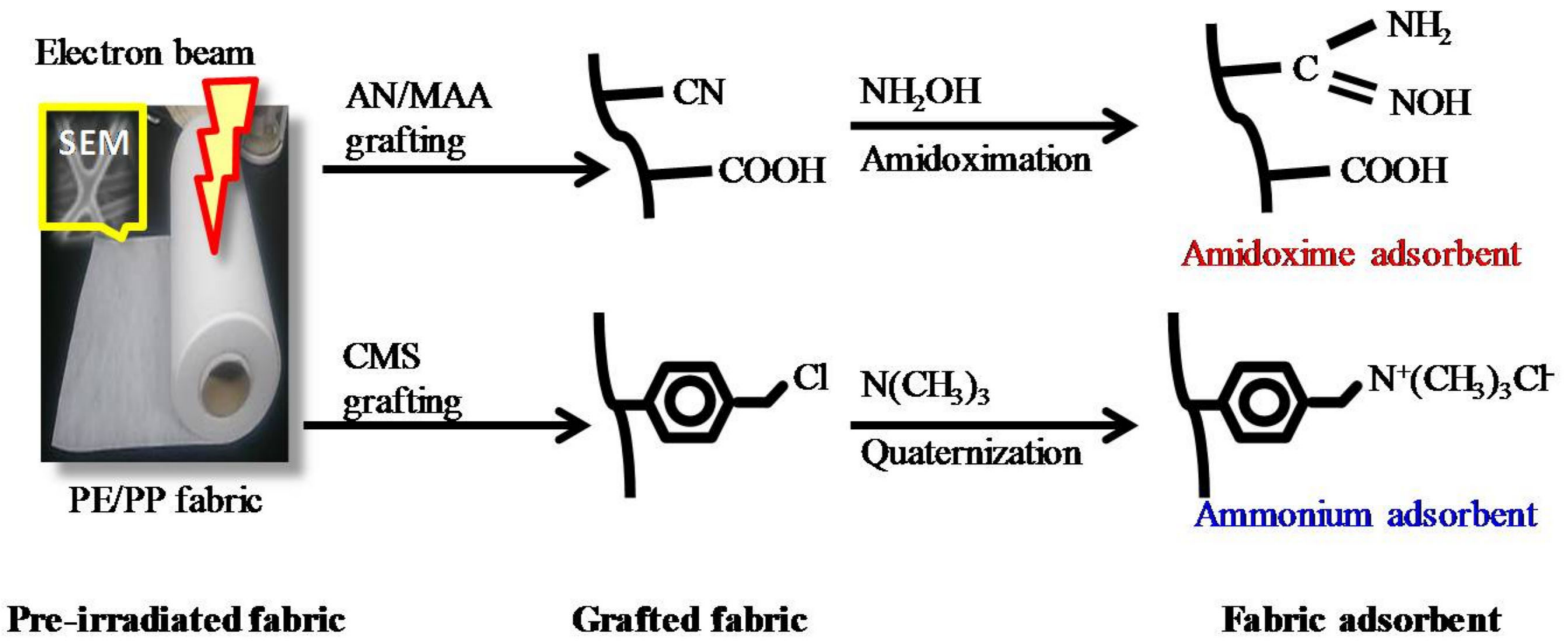
Process for the preparation of the amidoxime and ammonium fabric adsorbents.

Radiation grafting is a very attractive method for the modification of the existing polymeric materials, giving them special properties without losing their original characteristics [19,23,24,25,26,27,28,40]. The process for the preparation of the amidoxime and ammonium adsorbents is shown in Scheme 2, in which three steps are listed. First, the PE/PP nonwoven fabric was pre-irradiated at room temperature in a vacuum packed polyethylene bag. Under the irradiation, the radicals were generated on the PE/PP fabric due to the hydrogen abstraction and chain scission of the polymer [35]. The irradiated fabric was then immersed into a nitrogen-bubbled monomer solution in a glass ampoule, in which the radicals acted as active sites for initiating the graft polymerization. In Scheme 2, both the graft polymerizations for AN/MAA and CMS were carried at 40 °C for 1–5 h. After the grafting, the thickness and the surface area of the grafted fabric increased.

Finally, the grafted fabrics were converted into fabric adsorbents by introducing functional groups. In this study, the AN/MAA-grafted fabric was amidoximated in a hydroxylamine solution at 80 °C for more than 15 min, in which the cyanide (CN) groups of the graft chain reacted with the hydroxylamine, yielding the amidoxime (C(NH_2_)=NOH) groups and resulting in the amidoxime chelating fabric adsorbent [36]. On the other hand, the CMS-grafted fabric was quaternized in the trimethylamine solution at 80 °C for more than 30 min, where the trimethylamine reacted with the chloromethyl (CH_2_Cl) groups on the graft chains, yielding the quaternary ammonium (N^+^(CH_3_)_3_Cl^−^) groups and hence resulting in the quaternary ammonium fabric adsorbent [38,39].

**Figure 2:**
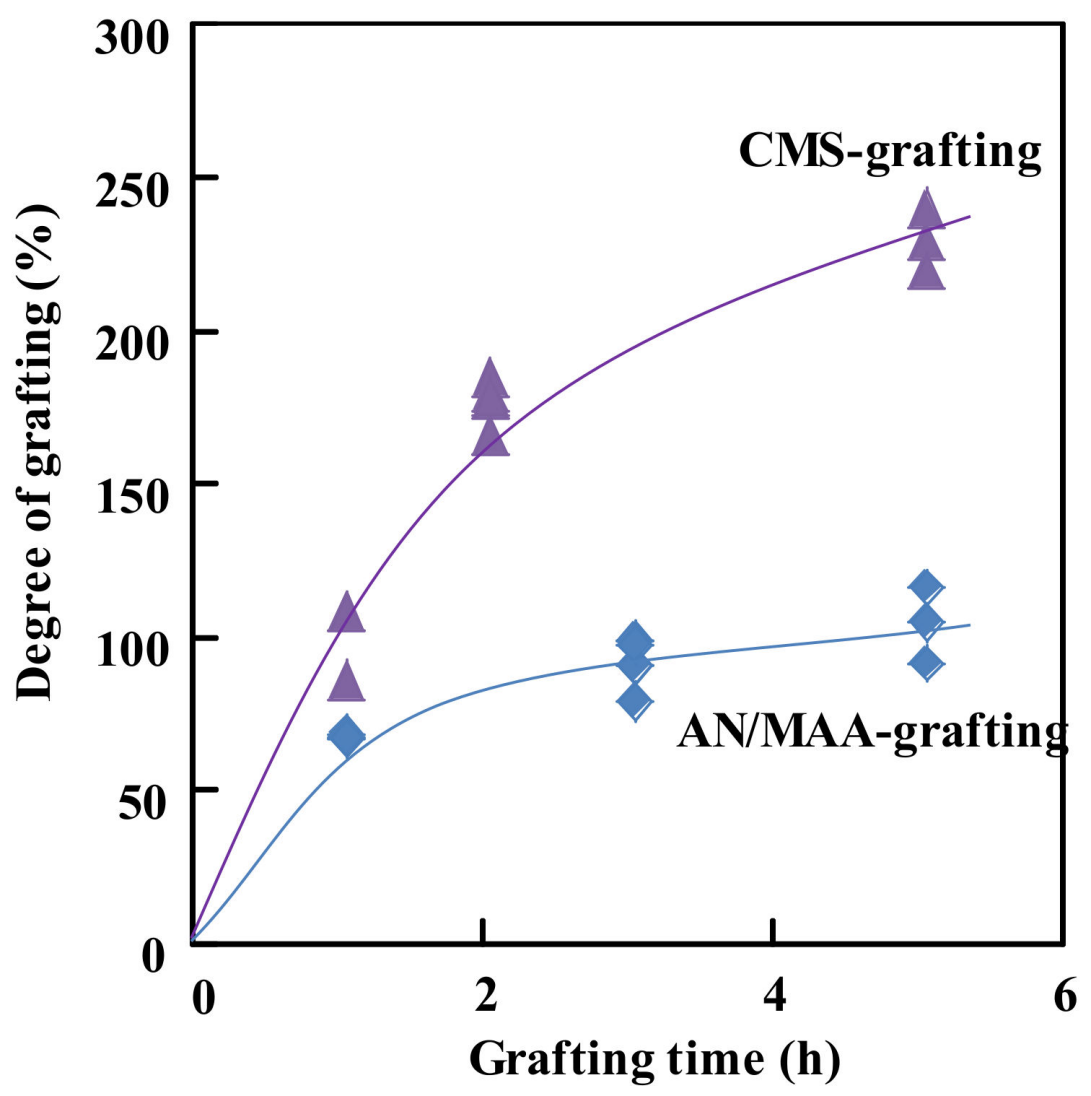
Radiation grafting of AN/MAA and CMS onto the PE/PP nonwoven fabrics in nitrogen gas. Preirradiation dose, 100 kGy for AN/MAA grafting and 50 kGy for CMS grafting; grafting temperature, 40 °C.

Figure 2 shows the kinetics of the radiation grafting of AN/MAA and CMS onto the PE/PP fabrics, respectively. The pre-irradiation dose for the grafting of AN/MAA was 100 kGy, and the AN/MAA/DMSO monomer solution (35/15/50 in the weight ratio) was used. The grafting was carried out at 40 °C under an oxygen-free environment. As shown in Figure 2, the degree of grafting initially increased, and then plateau out into a level of about 100%, indicating that the radicals were consumed after 2 h of the grafting.

On the other hand, for the CMS-grafting, the pre-irradiation dose was 50 kGy, and the monomer solution was an emulsion containing a low CMS concentration of 3.0 wt %. Even then, the grafting was significantly faster than the AN/MAA-grafting, that is, the degree of grafting increased to 100% within 1.0 h, and then continuously increased to 230% after 5.0 h. The fast and high grafting ability may be ascribed to the highly stable radicals in the solid fabrics in the emulsion system, where the radicals transfer rate from the fabric to the emulsion was slow due to the poor interface affinity. On the other hand, in the AN/MAA/DMSO system, the good interface affinity caused the rapid transfer of the radicals to the solution, resulting in the quicker radical consumption and the lower degree of grafting.

The AN/MAA- and CMS-grafted fabrics were amidoximated and quaternized by amidoxime and trimethylamine, respectively, to obtain the relevant fabric adsorbents. These reactions rendered the grafted fabrics high affinity to heavy metal ions as well as highly hydrophilic in nature. The suitable solvent was methanol/water in the weight ratio of 1/1, as it was well miscible with the reaction agents and had a high affinity with the grafted fabrics. Excess hydroxylamine and trimethylamine were used to ensure the adequate reaction of the grafted fabrics.

At the high temperature of 80 °C, both the reactions of amidoximation and quaternization were quickly processed. In the preliminary experiment, we confirmed that all the cyanide (CN) groups in the AN/MAA-grafted fabrics were amidoximated within 15 min, and all the chloromethyl (CH_2_Cl) groups in the CMS-grafted fabrics were quaternized within 30 min. The co-grafted MAA in the grafted fabric enhanced the amidoximation due to their hydrophilicity [36].

**Figure 3:**
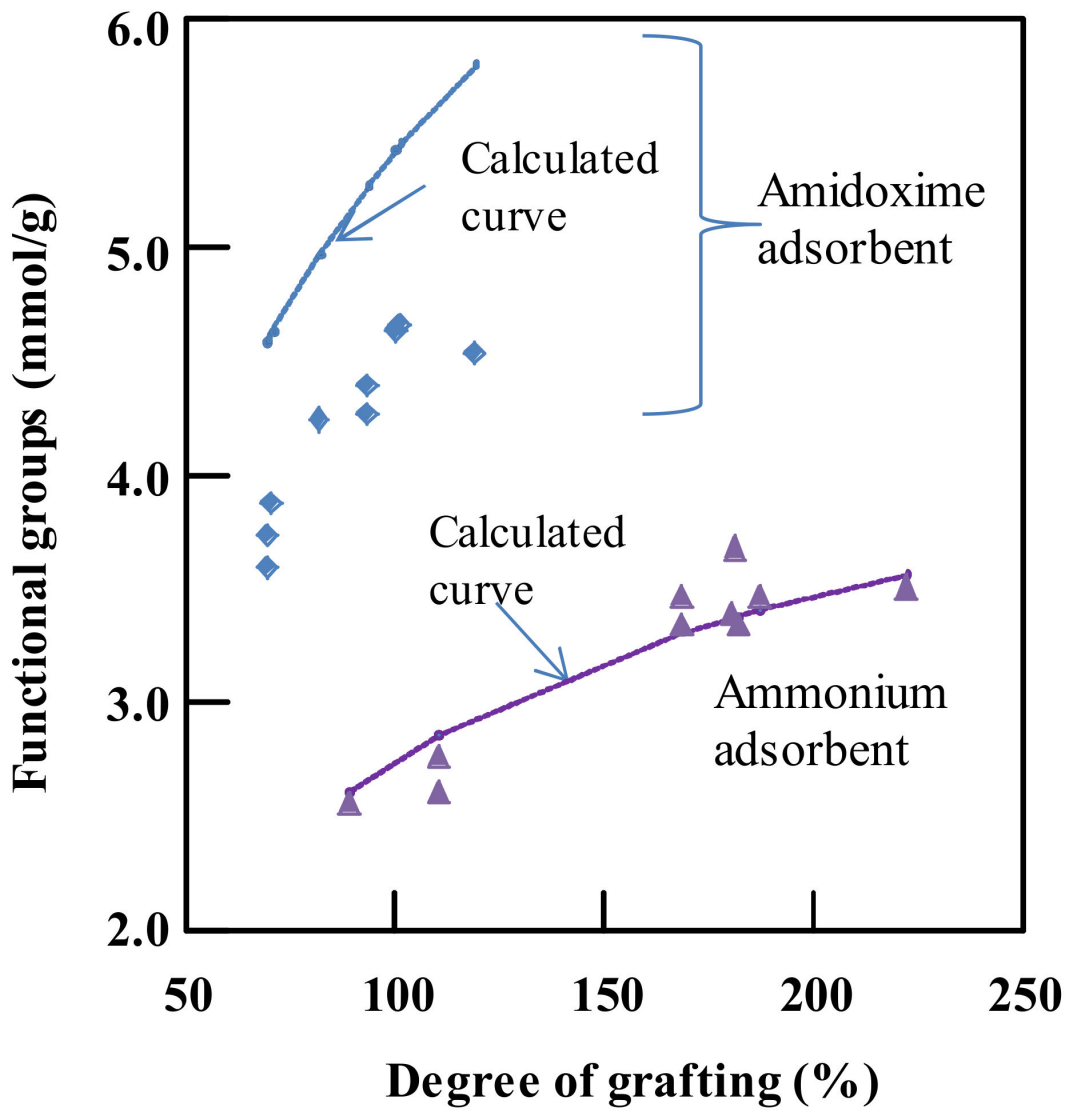
Functional groups in the amidoxime and ammonium adsorbents as a function of the degree of grafting. The amidoxime adsorbent is obtained by immersing the AN/MAA-grafted fabric in a hydroxylamine solution at 80 °C for more than 15 min; and the ammonium adsorbent is obtained by immersing the CMS-grafted fabric in a trimethylamine solution at 80 °C for more than 30 min.

Figure 3 shows the density of functional groups, amidoxime and quaternary ammonium groups, in the relevant adsorbents as a function of the degree of grafting. The calculated curves were obtained by assuming each active site attached by one functional group. Here, the mole percentage of the AN unit in the graft chains was supposed to be equal to that in the monomer solution [41]. The detected density of functional groups from CMS-grafted fabrics was in good agreement with the calculated data. However, for the AN/MAA-grafted fabric adsorbents, the detected values were quite lower than the calculated ones. The AN unit in the graft chains, estimated from the introduced amidoxime groups, was about 50 mol %, which was lower than 70 mol % in the monomer solution. Even then, the amidoxime adsorbent showed a high density of functional groups of 4.53 mmol/g when the degree of grafting was 119%, while for the CMS-grafted fabric adsorbents, the density of the ammonium groups reached 3.51 mmol/g when the degree of grafting was 222%. The density of the functional groups in the fabric adsorbents was directly related to the adsorption ability. As shown in Figure 3, the density of functional groups of the adsorbent could be controlled accordingly by changing the degree of grafting. With a higher degree of grafting, a higher degree of functional groups and thus higher ability of ion adsorption could be expected. In this work, the amidoxime adsorbent and the ammonium adsorbent with high densities of functional group of 4.53 and 3.51 mmol/g, respectively, were used in the subsequent adsorption tests, because they could possibly have high chromium removal and the adequate mechanical strength for actual applications [41].

### 3.2. Characterization of Fabric Adsorbents by FTIR/ATR and TG/DTA Analysis

**Figure 4:**
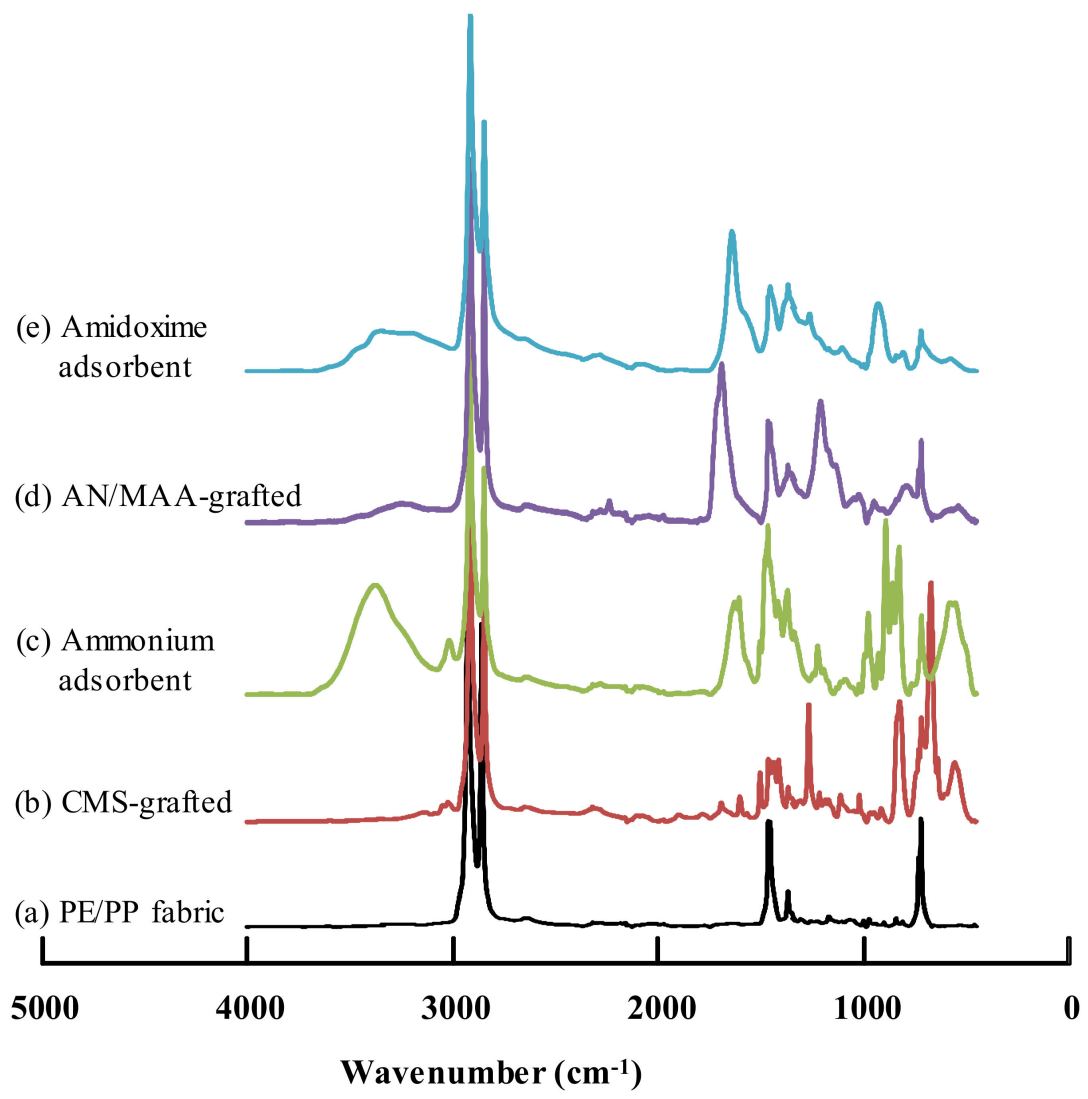
FTIR/ATR spectra for (**a**) the PE/PP nonwoven fabric; (**b**) the CMS-grafted fabric with a degree of grafting of 222%; (**c**) the relevant ammonium adsorbent; (**d**) the AN/MAA-grafted fabric with a degree of grafting of 119%; and (**e**) the relevant amidoxime adsorbent.

Figure 4 shows the typical FTIR/ATR spectra of (a) original PE/PP fabric, (b) CMS-grafted fabric with a degree of grafting of 222%, (c) the relevant adsorbent with a density of ammonium groups of 3.51 mmol/g, (d) AN/MAA-grafted fabric with a degree of grafting of 119%, and (e) the relevant adsorbent with a density of amidoxime group of 4.53 mmol/g. For the PE/PP fabric, only the typical FTIR spectrum for polyethylene was observed, that is, the antisymmetric and symmetric stretching vibrations, the bending vibration and the rocking vibration for polyethylene were 2916, 2848, 1465 and 726 cm^−1^, respectively, while the characteristic absorption bands for polypropylene, such as bending vibration of CH_3_ around 1380 cm^−1^, was not observed. These results indicated that the PP in the PE/PP fabric was completely coated by the PE, and thus could not be detected by the FTIR in the ATR mode.

After the CMS-grafting with a degree of grafting of 222%, the appearance of strong peaks at 1266, 789 and 673 cm^–1^ was ascribed to C–Cl stretching vibration of the chloromethyl groups [42], and the peaks around 1470 and 3030 cm^–1^ were ascribed to the aromatic ring. After reaction with trimethylamine at 80 °C for 15 min, the quaternized fabrics showed new characteristic peaks at 1503 and 1222 cm^–1^, attributed to the quaternary ammonium cations, while the peaks at 1266 and 673 cm^−1^ derived from C–Cl bond disappeared, indicating that the chloromethyl groups were completely quaternized, and the ammonium adsorbent was successfully prepared. The ammonium adsorbent was hydrophilic, and the peaks around 3400 and 1650 cm^−1^ were due to the adsorbed water.

On the other hand, after the AN/MAA-grafting, a small peak at 2249 cm^−1^ for the cyanide groups and a strong peak at 1702 cm^−1^ for the carboxyl groups were observed. After reaction with amidoxime, the small peak at 2249 cm^−1^ disappeared. The new absorption bands at 3200–3500, 1378 and 938 cm^−1^ were attributed to the amidoxime groups. These results indicated that the AN/MAA was co-grafted onto the PE/PP fabric, and the grafted fabric was successfully amidoximated with hydroxylamine. Thus, the amidoxime adsorbent was obtained.

**Figure 5:**
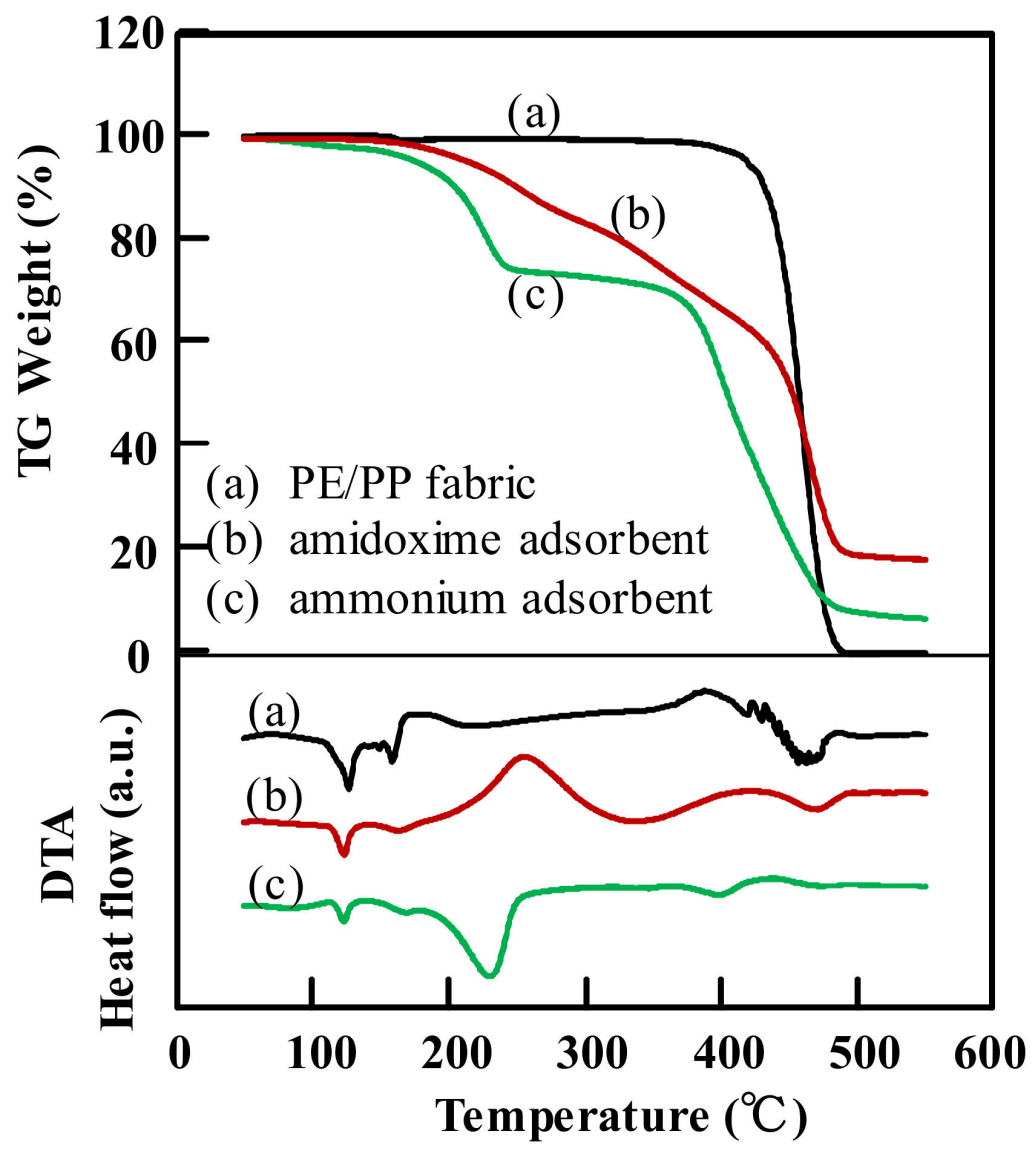
TG/DTA analysis for (a) the PE/PP nonwoven fabric; (b) the amidoxime adsorbent with a density of functional groups of 4.53 mmol/g; and (c) the ammonium adsorbent with a density of functional groups of 3.51 mmol/g.

The TG/DTA curves of amidoxime and ammonium adsorbents as well as the original PE/PP fabric are shown in Figure 5. The original PE/PP fabric showed high thermal stability up to 390 °C, above which it decomposed quickly. The observed endothermic peaks on the DTA curve, attributed to the melting points of the PE and PP, were around 125 and 160 °C, respectively.

For the amidoxime adsorbent, the TG curve showed a gradual weight decrease in the range of 165–438 °C. The weight loss was due to the complex thermal decomposition among the amidoxime and carboxylic groups, and the graft chains. On the contrary, for the ammonium adsorbent, the TG curve showed two-step weight loss which occurred in the temperature range of 150–250 °C and above the temperature 380 °C. The former was due to the degradation of the quaternary ammonium groups on the graft chains, and the latter was due to the degradation of the graft chains as well as the trunk polymer. The thermal changes during the degradation reactions could be confirmed on the relevant DTA curves. Furthermore, for the TG curve of amidoxime adsorbent, the weight loss in the temperature range of 150–250 °C was close to the calculated value, assuming that all the quaternary ammonium groups were decomposed. On the other hand, as shown on the DTA curves, the melting peaks for the PE and PP did not disappear after the modifications, indicating that the intrinsic crystallinity of the PE/PP fabric was unchanged during the grafting and the chemical functionalization, which in turn implied that the grafting took place primarily in the amorphous region, or on the surface of the crystal particles of the polymer. Therefore, both the amidoxime and ammonium adsorbents were stable up to 150 °C, below which the functional groups were not decomposed and the adsorbents could be used for heavy ions removal.

### 3.3. Batch Mode Adsorption

For the batch mode adsorption tests, the adsorbents (about 20 mg) were immersed in 45 mL of a 0.1 ppm chromium solution. The pH was adjusted using HNO_3_ and NaOH. Here, the stoichiometry of the adsorbent was much greater than that of the chromium in the solution. The pH strongly affected the forms of the chromium ions and the surface charge of the adsorbents. The adsorption tests were carried out for the chromium solution at the low pH 2.0 and the high pH 5.0.

**Figure 6:**
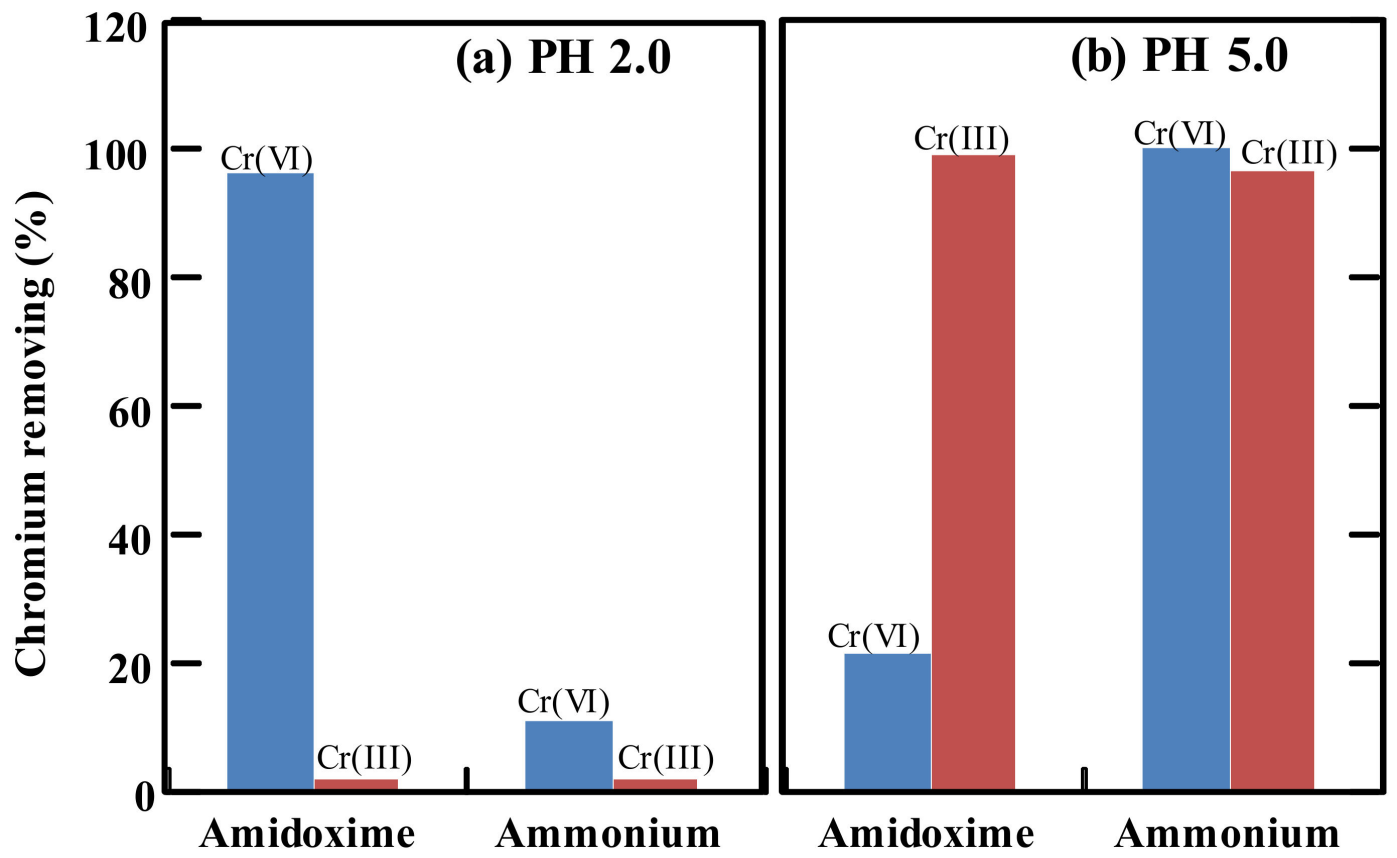
Chromium removing by amidoxime and ammonium adsorbents in the batch mode adsorption at (**a**) pH 2.0 and (**b**) pH 5.0. Initial chromium solution, 0.1 ppm; volume, 45 mL; density of functional groups, 4.53 mmol/g for the amidoxime adsorbent and 3.51 mmol/g for the ammonium adsorbent; adsorption time, 90 min; room temperature.

At the low pH 2.0, the Cr(VI) and Cr(III) were in the form of HCrO_4_^−^ and Cr^3+^, respectively. Herein, the nitrogen element in the adsorbent having free electron doublets was well protonated due to the strong acid environment, resulting in the relatively positive charge on the adsorbent surface. Therefore, the Cr(III) in the form of Cr^3+^ was difficult to be adsorbed due to the strong positive electric repulsion. As shown in Figure 6a, both the amidoxime adsorbent and the ammonium adsorbent showed a low percentage of Cr(III) removing. On the contrary, the Cr(VI) in the form of HCrO_4_^−^ was negative charged, and easily got close to the protonated amidoxime group for chelating and adsorption. As a result, the amidoxime adsorbent showed a high Cr(VI) removing percentage close to 100%. However, the ammonium adsorbent could not remove the Cr(VI) anions in the same system. This was due to the large amount of competing nitric anions in the solution at the low pH 2.0. The adsorption of Cr(VI) anions by the ammonium adsorbent was an anion exchange process. The nitric anions with a much higher concentration were preferentially adsorbed on the active sites, and thus decreased the Cr(VI) adsorption. Therefore, the condition under the low pH 2.0 was not suitable for the Cr(VI) removal by ammonium adsorbents. In other words, the solution with low pH may be used as an eluent for desorption of the Cr(VI) on the adsorbent.

On the other hand, at the high pH 5.0, the Cr(VI) was in the forms of HCrO_4_^−^ and CrO_4_^2−^, while the Cr(III) was in the forms of Cr^3+^, Cr(OH)^2+^ and Cr(OH)_2_^+^. The anion concentration was relatively low in the solution. The amidoxime groups on the adsorbents were almost not protonated. In this case, as shown in Figure 6b, the Cr(VI) could not be adsorbed by the amidoxime adsorbents, showing a low Cr(VI) removing percentage. On the contrary, the Cr(III) could be largely removed by the amidoxime adsorbents, reaching a high removing close to 100%. This may be due to the ligand formation between the Cr(III) and the amidoxime groups, and the ion bond between the Cr(III) and carboxylic groups at the high pH. On the other hand, the ammonium adsorbents could effectively remove both the Cr(III) and the Cr(VI) in the solution at pH 5.0. The Cr(VI) in the anion form was removed according to the anion exchange mechanism, while the Cr(III) in the cation form may be removed according to a physical adsorption. Therefore, the condition under the pH 5.0 was more suitable for the chromium removal, under which both the Cr(III) and the Cr(VI) could be removed by ammonium adsorbents, and the Cr(III) could be also removed by the amidoxime adsorbents to a large extent.

### 3.4. Column Mode Adsorption

As mentioned above, the ammonium adsorbent could completely remove the Cr(III) and Cr(VI) in the water at pH 5.0. To further test the adsorbent, a column mode adsorption experiment was designed. The ammonium adsorbent was filled in a column, which was flowed by chromium water at a rate of 60 mL/h. The Cr(III) concentration in the inflow solution was 0.1 ppm, and the Cr(VI) concentration in the inflow solution was 10 ppm.

**Figure 7:**
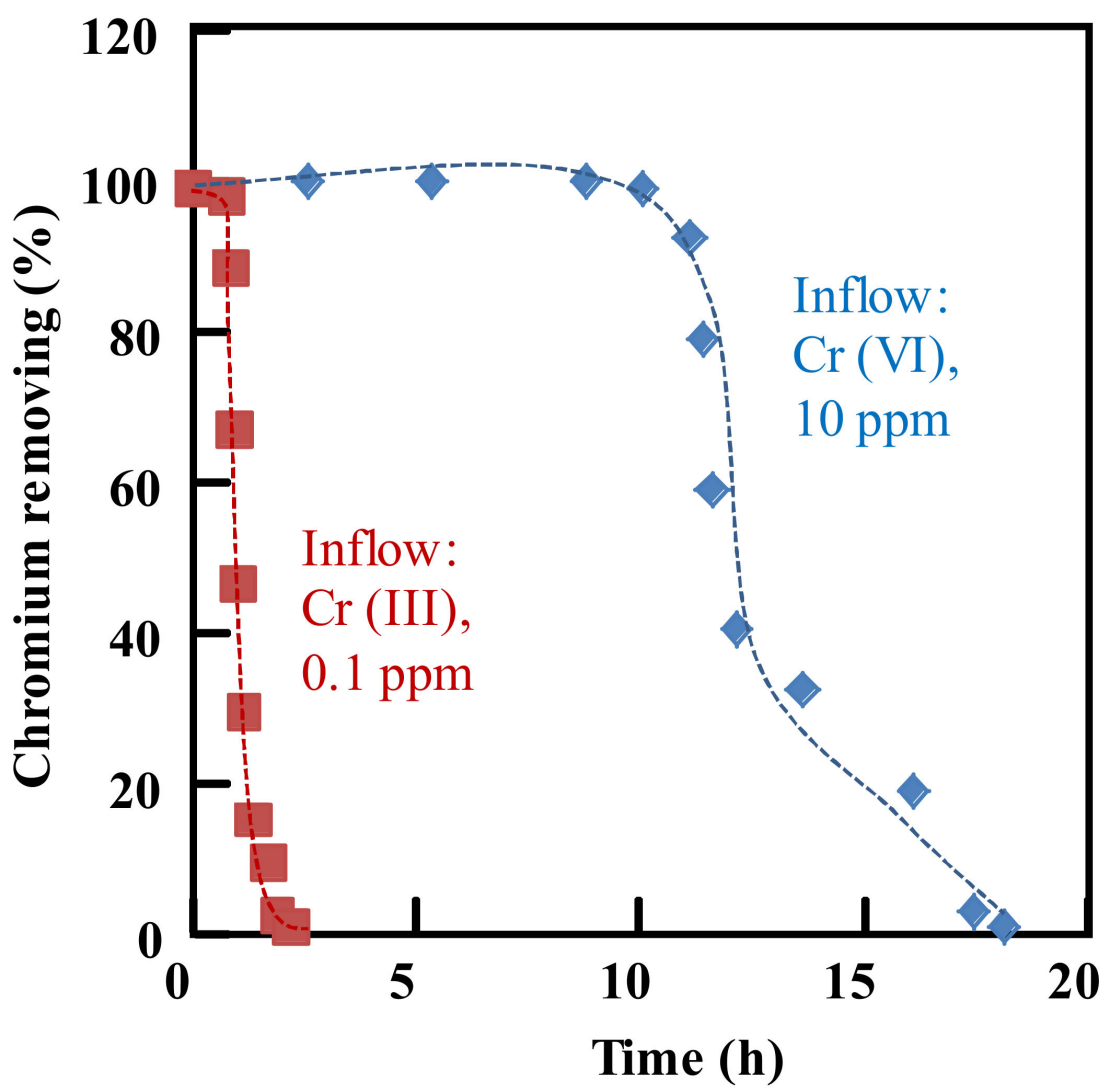
Chromium removing by the ammonium adsorbent in the column mode adsorption at pH 5.0. Density of functional groups, 3.51 mmol/g; inflow concentration, 0.1 ppm for Cr(III) and 10 ppm for Cr(VI); flow rate, 60 mL/h; room temperature.

Although the Cr(III) concentration (0.1 ppm) in the inflow solution was very low, as shown in Figure 7, the Cr(III) was soon detected in the outflow solution. The Cr(III) adsorption capacity of the adsorbents in the column, calculated from Figure 7 using Equation (4), was 0.12 mg/g. The low Cr(III) adsorption capacity of the ammonium adsorbents was due to the weak physical adsorption process. On the contrary, when the Cr(VI) solution was fed, the Cr(VI) ions could be removed up to a large outflow volume more than 600 mL (10 h of flow time), showing a high Cr(VI) adsorption capacity of 130.65 mg/g. The high Cr(VI) adsorption capacity of the ammonium adsorbents was due to the excellent anion exchange between the Cr(VI) anions and the adsorbents. Theoretically, in the case of a full anion exchange between the adsorbents and the Cr(VI) ions, the calculated Cr(VI) adsorption capacity of the tested ammonium adsorbents with an anion exchange capacity of 3.51 mmol/g was 182 mg/g. The relatively low value of the actual adsorption capacity was due to the presence of the competing anions in the solution.

**Figure 8:**
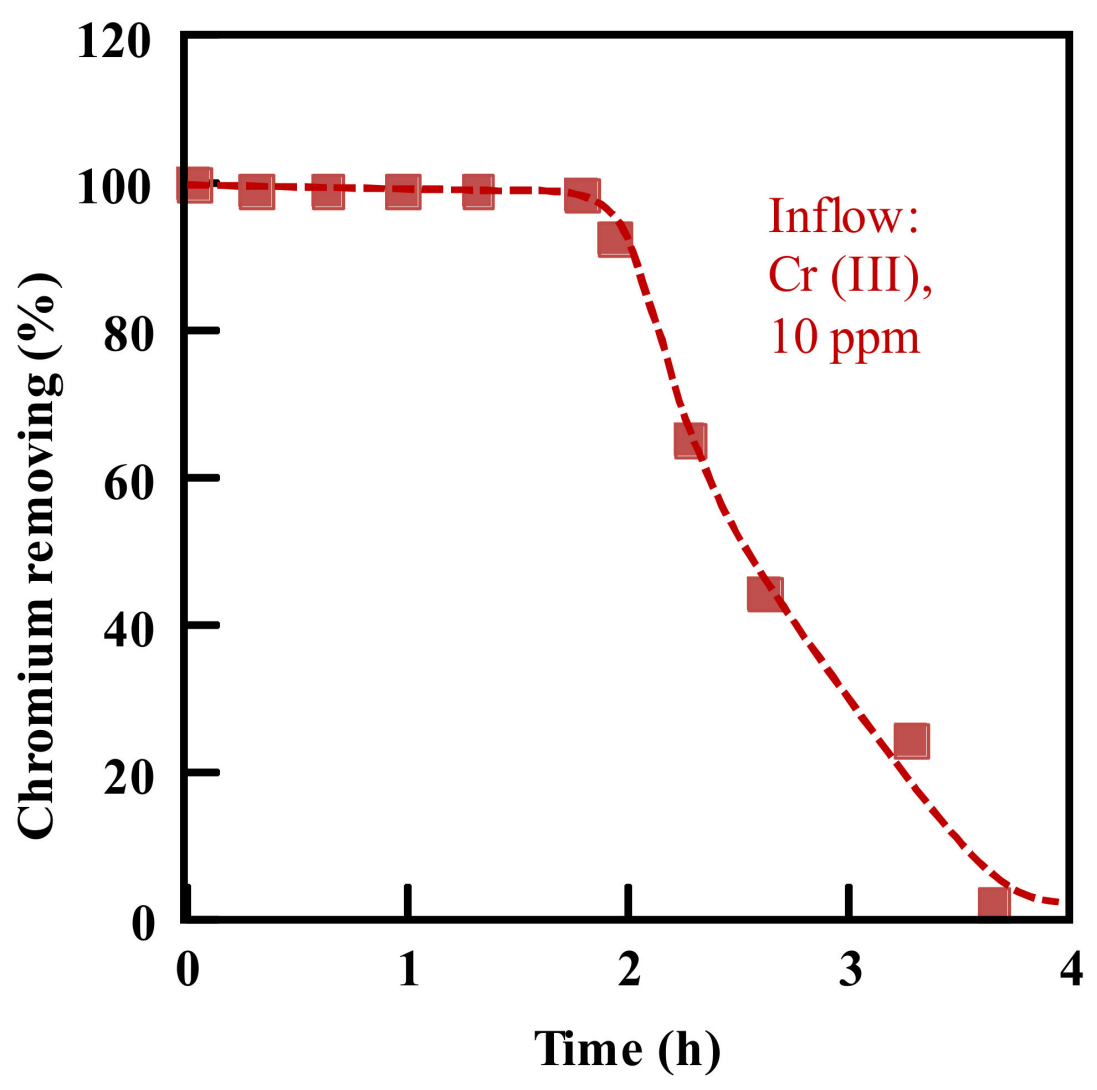
Chromium removing by the amidoxime adsorbent in the column mode adsorption at pH 5.0. Density of functional groups, 4.53 mmol/g; inflow solution, 10 ppm for the Cr(III); flow rate, 60 mL/h; room temperature.

To remove the Cr(III) from water, a similar test was performed using the amidoxime adsorbents. Figure 8 showed the relevant column mode adsorption results. In this case, it was found that the amidoxime fabric adsorbent removed the Cr(III) with a volume up to 120 mL after 2 h of flow time, showing a reasonable Cr(III) adsorption capacity of 31.68 mg/g. On the contrary, when the Cr(VI) solution was fed, its concentration in the outflow solution was almost not changed even at the beginning, indicating that the amidoxime adsorbent could not adsorb the Cr(VI) ions.

Furthermore, both the ammonium and amidoxime adsorbents could be easily regenerated by 1.0 M sodium hydroxide, where the adsorbed chromium was completely eluted into the sodium hydroxide solution, so that the metal ions could be recovered and the adsorbents could be reused.

## 4. Conclusions

Two types of nitrogen-containing fabric adsorbents, amidoxime and ammonium adsorbents, were successfully prepared by radiation grafting. The amidoxime adsorbent was prepared by AN/MAA-grafting and subsequent amidoximation with hydroxylamine, while the ammonium adsorbent was obtained by grafting of CMS followed by quaternization with trimethylamine. The FTIR/ATR results confirmed the graft chains, and verified the functional groups were distributed in the resultant adsorbents. The TG/DTA results indicated that thermal stability of the prepared adsorbents was adequate for applications.

At the low pH 2.0, the amidoxime adsorbent could remove the Cr(VI) ions, while the ammonium adsorbent could barely remove the Cr(VI) or Cr(III) ions. On the contrary, at high pH 5.0, the amidoxime adsorbent could remove the Cr(III) ions, and the ammonium adsorbent could remove both the Cr(III) and Cr(VI) ions. Therefore, the Cr(VI) and Cr(III) ions in the wastewater at pH 5.0 were possible to be completely removed by the prepared adsorbents. In the column mode adsorption, it was found that the Cr(III) adsorption capacity of the amidoxime adsorbent reached a high level of 31.68 mg/g, and the Cr(VI) adsorption capacity of the ammonium adsorption reached a much higher level of 130.65 mg/g. Furthermore, the adsorbed chromium could be desorbed by rinsing with the sodium hydroxide solution, and the adsorbents could be repeatedly reused.

## References

[1] Costa M., Klein C.B. (2006). Toxicity and carcinogenicity of chromium compounds in humans. Crit. Rev. Toxicol..

[2] Golbaz S., Jafari A.J., Rafiee M., Kalantary R.R. (2014). Separate and simultaneous removal of phenol, chromium, and cyanide from aqueous solution by coagulation/precipitation: Mechanisms and theory. Chem. Eng. J..

[3] Martín-Domínguez A., Rivera-Huerta M.D.L., Pérez-Castrejón S., Garrido-Hoyos S.E., Villegas-Mendoza I.E., Gelover-Santiago S.L., Drogui P., Buelna G. (2018). Chromium removal from drinking water by redox-assisted coagulation: Chemical versus electrocoagulation. Sep. Purif. Technol..

[4] Duan W., Chen G., Chen C., Sanghvi R., Iddya A., Walker S., Liu H., Ronen A., Jassby D. (2017). Electrochemical removal of hexavalent chromium using electrically conducting carbon nanotube/polymer composite ultrafiltration membranes. J. Membr. Sci..

[5] Dharnaik A.S., Ghosh P.K. (2014). Hexavalent chromium [Cr(VI)] removal by the electrochemical ion-exchange process. Environ. Technol..

[6] Rengaraj S., Yeon K.H., Moon S.H. (2001). Removal of chromium from water and wastewater by ion exchange resins. J. Hazard. Mater..

[7] Dehghani M.H., Sanaei D., Ali I., Bhatnagar A. (2016). Removal of chromium (VI) from aqueous solution using treated waste newspaper as a low-cost adsorbent: Kinetic modeling and isotherm studies. J. Mol. Liq..

[8] Habiba U., Siddique T.A., Joo T.C., Salleh A., Ang B.C., Afifi A.M. (2017). Synthesis of chitosan/polyvinyl alcohol/zeolite composite for removal of methyl orange, Congo red and chromium (VI) by flocculation/adsorption. Carbohydr. Polym..

[9] Fu F., Wang Q. (2011). Removal of heavy metal ions from wastewaters: A review. J. Environ. Manag..

[10] Khezami L., Capart R. (2005). Removal of chromium (VI) from aqueous solution by activated carbons: Kinetic and equilibrium studies. J. Hazard. Mater..

[11] Gupta V.K., Agarwal S., Saleh T.A. (2011). Chromium removal by combining the magnetic properties of iron oxide with adsorption properties of carbon nanotubes. Water Res..

[12] Garg U.K., Kaur M.P., Garg V.K., Sud D. (2007). Removal of hexavalent chromium from aqueous solution by agricultural waste biomass. J. Hazard. Mater..

[13] Gu H., Rapole S.B., Sharma J., Huang Y., Cao D., Colorado H.A., Luo Z., Haldolaarachchige N., Yong D., Walters B. (2012). Magnetic polyaniline nanocomposites toward toxic hexavalent chromium removal. RSC Adv..

[14] Kampalanonwat P., Supaphol P. (2010). Preparation and adsorption behavior of aminated electrospun polyacrylonitrile nanofiber mats for heavy metal ion removal. ACS Appl. Mater. Int..

[15] Qiu B., Xu C., Sun D., Wei H., Zhang X., Guo J., Wei S. (2014). Polyaniline coating on carbon fiber fabrics for improved hexavalent chromium removal. RSC Adv..

[16] Mayer-Gall T., Opwis K., Gutmann J.S. (2015). Polyvinylamine modified polyester fibers–innovative textiles for the removal of chromate from contaminated groundwater. J. Mater. Chem. A.

[17] Wang J., Pan K., Giannelis E., Bao B. (2013). Polyacrylonitrile/polyaniline core/shell nanofiber mat for removal of hexavalent chromium from aqueous solution: Mechanism and applications. RSC Adv..

[18] Barsbay M., Kavaklı P.A., Güven O. (2010). Removal of phosphate using copper-loaded polymeric ligand exchanger prepared by radiation grafting of polypropylene/polyethylene (PP/PE) nonwoven fabric. Radiat. Phys. Chem..

[19] Nasef M.M., Güven O. (2012). Radiation-grafted copolymers for separation and purification purposes: Status, challenges and future directions. Prog. Polym. Sci..

[20] Goel N.K., Kumar V., Misra N., Varshney L. (2015). Cellulose based cationic adsorbent fabricated via radiation grafting process for treatment of dyes waste water. Carbohydr. Polym..

[21] Verma S.K., Kaur I. (2012). Gamma-induced polymerization and grafting of a novel phosphorous-, nitrogen-, and sulfur-containing monomer on cotton fabric to impart flame retardancy. J. Appl. Polym. Sci..

[22] Li C., Zhang Y., Peng J., Wu H., Li J., Zhai M. (2012). Adsorption of Cr(VI) using cellulose microsphere-based adsorbent prepared by radiation-induced grafting. Radiat. Phys. Chem..

[23] Dargaville T.R., George G.A., Hill D.J., Whittaker A.K. (2003). High energy radiation grafting of fluoropolymers. Prog. Polym. Sci..

[24] Chen J., Asano M., Maekawa Y., Yoshida M. (2008). Chemically stable hybrid polymer electrolyte membranes prepared by radiation grafting, sulfonation, and silane-crosslinking techniques. J. Polym. Sci. A Polym. Chem..

[25] Chen J., Asano M., Yamaki T., Yoshida M. (2005). Preparation of sulfonated crosslinked PTFE-graft-poly(alkyl vinyl ether) membranes for polymer electrolyte membrane fuel cells by radiation processing. J. Membr. Sci..

[26] Chen J., Asano M., Yamaki T., Yoshida M. (2006). Preparation and characterization of chemically stable polymer electrolyte membranes by radiation-induced graft copolymerization of four monomers into ETFE films. J. Membr. Sci..

[27] Chen J., Septiani U., Asano M., Maekawa Y., Kubota H., Yoshida M. (2007). Comparative study on the preparation and properties of radiation-grafted polymer electrolyte membranes based on fluoropolymer films. J. Appl. Polym. Sci..

[28] Chen J., Maekawa Y., Asano M., Yoshida M. (2007). Double crosslinked polyetheretherketone-based polymer electrolyte membranes prepared by radiation and thermal crosslinking techniques. Polymer.

[29] Saito T., Brown S., Chatterjee S., Kim J., Tsouris C., Mayes R.T., Kuo L., Oyola Y., Hanke C., Dai S. (2014). Uranium recovery from seawater: Development of fiber adsorbents prepared via atom-transfer radical polymerization. J. Mater. Chem. A.

[30] Liu X., Liu H., Ma H., Cao C., Yu M., Wang Z., Deng B., Wang M., Li J. (2012). Adsorption of the uranyl ions on an amidoxime-based polyethylene nonwoven fabric prepared by preirradiation-induced emulsion graft polymerization. Ind. Eng. Chem. Res..

[31] Biniak S., Pakuła M., Szymański G.S., Światkowski A. (1999). Effect of activated carbon surface oxygen-and/or nitrogen-containing groups on adsorption of copper (II) ions from aqueous solution. Langmuir.

[32] Li Q., Qian Y., Cui H., Zhang Q., Tang R., Zhai J. (2011). Preparation of poly(aniline-1,8-diaminonaphthalene) and its application as adsorbent for selective removal of Cr(VI) ions. Chem. Eng. J..

[33] Zhao J., Zhang X., He X., Xiao M., Zhang W., Lu C. (2015). A super biosorbent from dendrimer poly(amidoamine)-grafted cellulose nanofibril aerogels for effective removal of Cr(VI). J. Mater. Chem. A.

[34] Huang S.H., Chen D.H. (2009). Rapid removal of heavy metal cations and anions from aqueous solutions by an amino-functionalized magnetic nano-adsorbent. J. Hazard. Mater..

[35] Fel E., Khrouz L., Massardier V., Cassagnau P., Bonneviot L. (2016). Comparative study of gamma-irradiated PP and PE polyolefins part 2: Properties of PP/PE blends obtained by reactive processing with radicals obtained by high shear or gamma-irradiation. Polymer.

[36] Kawai T., Saito K., Sugita K., Kawakami T., Kanno J., Katakai A., Seko N., Sugo T. (2000). Preparation of hydrophilic amidoxime fibers by cografting acrylonitrile and methacrylic acid from an optimized monomer composition. Radiat. Phys. Chem..

[37] Ueki Y., Mohamed N.H., Seko N., Tamada M. (2011). Rapid biodiesel fuel production using novel fibrous catalyst synthesized by radiation-induced graft polymerization. Int. J. Org. Chem..

[38] Varcoe J.R., Slade R.C., Lam How Yee E., Poynton S.D., Driscoll D.J., Apperley D.C. (2007). Poly(ethylene-*co*-tetrafluoroethylene)-derived radiation-grafted anion-exchange membrane with properties specifically tailored for application in metal-cation-free alkaline polymer electrolyte fuel cells. Chem. Mater..

[39] Kawai T., Saito K., Sugita K., Katakai A., Seko N., Sugo T., Kanno J., Kawakami T. (2000). Comparison of amidoxime adsorbents prepared by cografting methacrylic acid and 2-hydroxyethyl methacrylate with acrylonitrile onto polyethylene. Ind. Eng. Chem. Res..

[40] Chen J., Seko N. (2017). Effects of RAFT Agent on the Chloromethylstyrene Polymerizations in a Simultaneous Radiation Grafting System. Polymers.

[41] Kavaklı P.A., Seko N., Tamada M., Güven O. (2007). Radiation-induced graft polymerization of glycidyl methacrylate onto PE/PP nonwoven fabric and its modification toward enhanced amidoximation. J. Appl. Polym. Sci..

[42] Vandiver M.A., Caire B.R., Pandey T.P., Li Y., Seifert S., Kusoglu A., Knauss D., Herring A., Liberatore M.W. (2016). Effect of hydration on the mechanical properties and ion conduction in a polyethylene-b-poly(vinylbenzyl trimethylammonium) anion exchange membrane. J. Membr. Sci..

